# Subclinical Agoraphobia Symptoms and Regional Brain Volumes in Non-clinical Subjects: Between Compensation and Resilience?

**DOI:** 10.3389/fpsyt.2018.00541

**Published:** 2018-11-28

**Authors:** Bianca Besteher, Letizia Squarcina, Robert Spalthoff, Marcella Bellani, Christian Gaser, Igor Nenadić, Paolo Brambilla

**Affiliations:** ^1^Department of Psychiatry and Psychotherapy, Jena University Hospital, Jena, Germany; ^2^IRCCS “E. Medea” Scientific Institute, Lecco, Italy; ^3^Department of Psychiatry, Azienda Ospedaliera Universitaria Integrata Verona, Verona, Italy; ^4^Department of Neurology, Jena University Hospital, Jena, Germany; ^5^Department of Psychiatry and Psychotherapy, Philipps Universität Marburg, Marburg, Germany; ^6^Department of Psychiatry and Psychotherapy, University Hospital Giessen and Marburg, Marburg, Germany; ^7^Center for Mind, Brain and Behavior (CMMB), Marburg, Germany; ^8^Department of Neurosciences and Mental Health, Fondazione IRCCS Ca' Granda Ospedale Maggiore Policlinico, University of Milan, Milan, Italy; ^9^Department of Psychiatry and Behavioural Neurosciences, UT Houston Medical School, Houston, TX, United States

**Keywords:** anxiety disorders, imaging, neuroanatomy, phobias, healthy subjects, voxel-based morphometry

## Abstract

**Background:** Symptoms of anxiety are present not only in panic disorder or other anxiety disorders, but are highly prevalent in the general population. Despite increasing biological research on anxiety disorders, there is little research on understanding subclinical or sub-threshold symptoms relating to anxiety in non-clinical community samples, which could give clues to factors relating to resilience or compensatory changes.

**Aims:**This study focused on brain structural correlates of subclinical anxiety/agoraphobia symptoms from a multi-center imaging study.

**Methods:** We obtained high-resolution structural T1 MRI scans of 409 healthy young participants and used the CAT12 toolbox for voxel-based morphometry (VBM) analysis. Subjects provided self-ratings of anxiety using the SCL-90-R, from which we used the phobia subscale, covering anxiety symptoms related to those of panic and agoraphobia spectrum.

**Results:** We found significant (*p* < 0.05, FDR-corrected) correlations (mostly positive) of cortical volume with symptom severity, including the right lingual gyrus and calcarine sulcus, as well as left calcarine sulcus, superior, middle, and inferior temporal gyri. Uncorrected exploratory analysis also revealed positive correlations with GMV in orbitofrontal cortex, precuneus, and insula.

**Conclusions:** Our findings show brain structural associations of subclinical symptoms of anxiety, which overlap with those seen in panic disorder or agoraphobia. This is consistent with a dimensional model of anxiety, which is reflected not only functionally but also on the structural level.

## Introduction

Modern psychiatry has experienced a paradigm shift from categorical classifications of mental illness to dimensional approaches, taking into account the clinical and neurobiological continuum of psychopathological symptoms. This approach is highly relevant for neurobiological research (1–4) as well as diagnosing and treating mental illness (5). Yet, there is little biological research to understand the full spectrum, including its subclinical or subthreshold part, where symptoms might be prevalent but not reaching the threshold to manifest disorders. This includes a spectrum covering minor symptoms, which might transiently present in clinically healthy subjects, or “subthreshold” symptom combinations accompanied by some clinical burden, but not reaching the threshold of a DSM/ICD-defined clinical phenotype. While there is no generally accepted distinction between “subclinical” and “subthreshold” symptoms, some recent studies have referred to “subthreshold” psychopathologies as those which (although not meeting clinical criteria) are closer to clinical states and potentially predictive of subsequent transition to clinical psychopathologies [for example: (6–9)]. For the purpose of the present study, we shall refer to subclinical symptoms as those not meting clinical thresholds, without necessarily exceeding levels seen in the general population, and without explicitly assuming a link to imminent clinical stages of disorders.

Considering models of risk and resilience, the neurobiological underpinnings of these changes might lead to a better understanding of compensatory changes, which lead to subclinical (as opposed to clinical) phenotypes. So far, however, few studies have explicitly addressed the problem of neural correlates of subclinical symptoms.

Anxiety and phobic symptoms are not only among the most prevalent symptoms in various psychopathologies, but anxiety disorders constitute a considerable proportion of mental disorders (10). Common anxiety disorders are panic disorder and agoraphobia, with typical sudden attacks of anxiety, and anxiety about being in places or situations from which escape might be difficult or embarrassing or in which help might not be available in case that escape is needed, respectively (American Psychiatric Association, Diagnostic and Statistical Manual of Mental Disorders, 5th ed., 2013). The 12-month prevalence of panic disorder is 1.8% in the adult, Caucasian population aged 18–65 and around 1.3% suffer from agoraphobia (11). Not only are these the most common and often comorbid anxiety disorders in patients (12). Single panic attacks or mild symptoms of agoraphobia also occur in non-clinical populations or during stressful life-events, such as during complicated grief reactions (13). Epidemiologic follow-up studies have shown that there might also be a prodrome to the development of severe anxiety disorders (next to depression or substance abuse), characterized by panic attacks or mild agoraphobic symptoms (14–17).

Among the most common anxiety disorders associated with phobic symptoms are panic disorders and agoraphobia. Several recent neuroimaging studies in patients with these disorders have shown brain structural alterations, such as increase of gray matter volume (GMV) in left insula, superior temporal gyrus, midbrain and pons, as well as GMV decrease in right anterior cingulate cortex (ACC) in patients with panic disorder (18). Patients with comorbid agoraphobia also exhibit bilaterally decreased amygdala and left parahippocampal volume (19). A meta-analysis covering social and generalized anxiety disorders reported GMV reductions in right anterior cingulate and precentral as well as left inferior frontal and middle temporal gyri in patients. Some of these findings overlap with results from functional neuroimaging studies revealing hyperactivation of the ventral striatum and insula when anticipating agoraphobia-specific situations in agoraphobic patients (20) or viewing visual cues related to anxiety (21). However, other studies have also identified functional changes in fusiform, lingual, and calcarine cortices in panic disorder with agoraphobia (22). While there is considerable heterogeneity across different disorders, a recent meta-analysis of structural imaging across anxiety disorders (23) has suggested that ACC and inferior frontal GMV reductions are prevalent and might constitute a point of neurobiological overlap. These findings also overlap with more general studies of human fear and anxiety, which have pointed out the relevance of the amygdala and ACC (24, 25).

Overall, this data is based on comparison of clinical populations with healthy controls, and thus little is known about subclinical anxiety symptoms (e.g., agoraphobic). Yet, for all we know, many healthy controls (lacking a concurrent psychiatric diagnosis) might experience symptoms of anxiety due to stressful life events or even as a prodrome to later agoraphobia. It is therefore unclear, which of the above findings relate to those representing a putative neurobiological continuum reflecting different degrees of anxiety, and which might relate to the expression of a defined clinical disease phenotype.

We assembled a large cohort of participants (recruited from the general population) for voxel-based morphometry (VBM) analysis of subclinical agoraphobic symptoms, hypothesizing alterations of GMV in the limbic system (e.g., amygdala), ACC to be correlated with symptoms. We performed a whole-brain analysis in order to also identify effects in areas not reported in classical case-control studies of anxiety disorders. This analysis builds on a previous study of ours (26), in which we used VBM in a sample of 177 non-clinical (psychiatrically healthy) subjects, correlating variation in gray matter with subclinical depression (general), anxiety, and somatisation subscales. Analyzing a larger multi-center cohort, our present study focuses on the “phobic” anxiety subscale of the SCL90R, which reflects subclinical symptoms close to the agoraphobia spectrum.

## Methods

### Participants

We included 409 healthy young adults consisting of three sub-samples. The participants were recruited as healthy controls for ongoing case-control studies in Jena, Germany, and Verona/Milano, Italy. The Jena sample of 318 subjects was divided in two sub-samples for methodological reasons, resulting in a Jena-1 sample of 177 persons and the Jena-2 sample with 141 persons scanned later on the scanner following an extensive hardware and software upgrade, which led us to treat these two subject groups as separate samples. The Jena-1 sample has previously been used for analyses of sub-clinical depressive and anxiety symptoms, although not for the scale used in this present study (26). The Verona-Milano sample consisted of 91 persons.

The 318 participants recruited in Jena gave written informed consent to a study protocol approved by the local Ethics Committee of Jena University Medical School, while the 91 participants recruited in Verona-Milano provided written informed consent to a study protocol approved by the Ethics Committee of the Azienda Ospedaliera Universitaria of Verona.

Inclusion criteria were ability to provide written informed consent and an age of 18 or above. Exclusion criteria were: current or previous psychiatric disorders (axis I), central nervous neurological disorders, traumatic brain injury with loss of consciousness, learning disability, or contraindication for MRI scans. As our study design was based on a dimensional model, we did not define symptom level thresholds, other than manifest clinical psychiatric conditions.

None of the participants had a present or history of DSM-IV axis I disorders, as determined by careful screening via phone (Jena samples) resp. by a modified interview derived from the SCID-IV non-patient version (SCID-NP) (Verona-Milano sample). They also had no history of major neurological and unmedicated internal medical conditions, as well as psychiatric history in first-degree relatives.

To exclude major cognitive impairment (IQ < 80), IQ was estimated using the MWT-B, a German language inventory similar to the NART (27) in the two Jena samples. All Verona-Milano participants met this criterion, but IQ was estimated using the Italian version of the Wechsler Adult Intelligence Scale—Revised (28, 29).

To assess subclinical occurrence of (agora)phobic symptoms, subjects completed SCL-90-R at the time of study. The SCL-90-R is a well established self-rating instrument to assess a broad range of psychopathological symptoms (30), and is one of the most widely used symptom questionnaire with over 1,000 published studies in many clinical and non-clinical samples (31). It consists of 90 items covering multiple domains of well-being and psychopathology, each to be rated on a 0–4 Likert-type scale, which can then be analyzed syndrome-wise with nine different scales. From these, we selected the “phobia” scale (scale 7), which consists of 7 items, mainly covering agoraphobic symptoms with emphasis on situations with low availability of help, and avoidance behavior. Dividing the cumulating value of the scale by the number of items we calculated the scale value (mean value), resulting in a range from 0 to 4. An overview of demographic and psychometric data is given in Table 1. In our pooled sample the mean scale value was 0.077, the data did not follow a normal distribution (Kolmogorov-Smirnov-Test with *p* < 0.05).

**Table 1:** Demographic and psychometric data on the three sub-samples.

	**Jena-1**	**Jena-2**	**Verona-Milano**
	**(*n* = 177)**	**(*n* = 141)**	**(*n* = 91)**
Mean age (*SD*)	29.8 (±8.93)	32.12 (±14.27)	29.13 (±7.7)
Age_range	20–60	19–73	18–62
Gender	83 f, 94 m	87 f, 54 m	55 f, 36 m
Mean IQ (SD)	106.23 (±11.5)	115.87 (±14.81)	122.39 (±8.49)
Mean SCL-90_phob scale value (SD)	0.09 (±0.19)	0.09 (±0.24)	0.05 (±0.09)
SCL-90_phob_range	0–1.14	0–2	0–0.43

*Overview of age, gender, IQ and SCL90-R aggression subscale values given as mean and standard deviation (SD). f, female, m, male*.

### Magnetic resonance imaging (MRI)

Subjects for the Jena-1 sample underwent high–resolution T1-weighted MRI on a 3 Tesla Siemens Tim Trio scanner (Siemens, Erlangen, Germany) using a standard quadrature head coil and an axial 3-dimensional magnetization prepared rapid gradient echo (MP-RAGE) sequence (TR 2,300 ms, TE 3.03 ms, α 9°, 192 contiguous sagittal slices, FoV 256 mm, voxel resolution 1 × 1 × 1 mm; acquisition time 5:21 min).

Participants of the Jena-2 sample were scanned on a Siemens Prisma fit system (Siemens, Erlangen, Germany), which was based on the above Siemens Tim Trio scanner, after undergoing a significant upgrade (both hardware and software). The structural scan was acquired with an MP-RAGE sequence with similar parameters (TR 2,300 ms, TE 2.07 ms, α 9°, 192 contiguous sagittal slices, FoV 256 mm, voxel resolution 1 × 1 × 1 mm; acquisition time 5:21 min) as part of a 25 min imaging session.

The Verona-Milano sample MRI scans were acquired with a 3T Magnetom Allegra Syngo MR 2004A (Siemens, Erlangen, Germany) also using a standard head coil for radio frequency transmission and reception of the MRI signal. An MP-RAGE sequence was acquired (TR 2,060 ms, TE 3.93 ms, α 15°, 160 contiguous sagittal slices, FoV 256 mm, voxel resolution 1 × 1 × 1 mm, acquisition time 7:32 min). The scan was part of a MRI protocol of about 50 min total duration.

All scans were checked to exclude imaging artifacts.

### Voxel-based morphometry

For VBM, we used the CAT 12 toolbox (Computational Anatomy Toolbox 12) of the Structural Brain Mapping group, Jena University Hospital, Jena, Germany (C. Gaser), which is implemented in SPM12 (Statistical Parametric Mapping, Institute of Neurology, London, UK). All T1-weighted images were corrected for bias—field inhomogeneities, then segmented into gray matter (GM), white matter (WM), and cerebrospinal fluid (CSF) (32) and spatially normalized using the DARTEL algorithm (33). The segmentation process was further extended by accounting for partial volume effects (34), applying adaptive maximum a posteriori estimations (35). After pre-processing and in addition to visual checks for artifacts all scans passed an automated quality check protocol. Scans were smoothed with a Gaussian kernel of 8 mm (FWHM). For exclusion of artifacts on the gray/white matter border (i.e., incorrect voxel classification), we applied an absolute gray matter threshold of 0.1.

### Statistics

For statistical comparison, we applied the general linear model (GLM) approach implemented in SPM12. For every analysis, we used scanner as a factor in order to eliminate the confounding effects of the three different scanner (setups) used. We performed one GLM correlating SCL-90-R phobia subscale values with GMV. For VBM- analysis we included total intracranial volume (TIV) as a nuisance variable in order to remove the related variance and applied correction for multiple comparisons at a threshold of *p* < 0.05, FDR-corrected. We performed whole-brain analyses investigating both positive and negative correlation between scale value and GMV.

In addition to this main analysis we performed an exploratory analysis (also for future hypothesis-generation purposes) at an uncorrected *p* < 0.001 threshold.

## Results

Cortical volume showed multiple (positive) correlations with the SCL-90-R phobia subscale in several clusters (*p* < 0.05, FDR-corrected), in particular right lingual gyrus and calcarine sulcus, as well as left calcarine sulcus, superior, middle and inferior temporal gyri.

**Figure 1:**
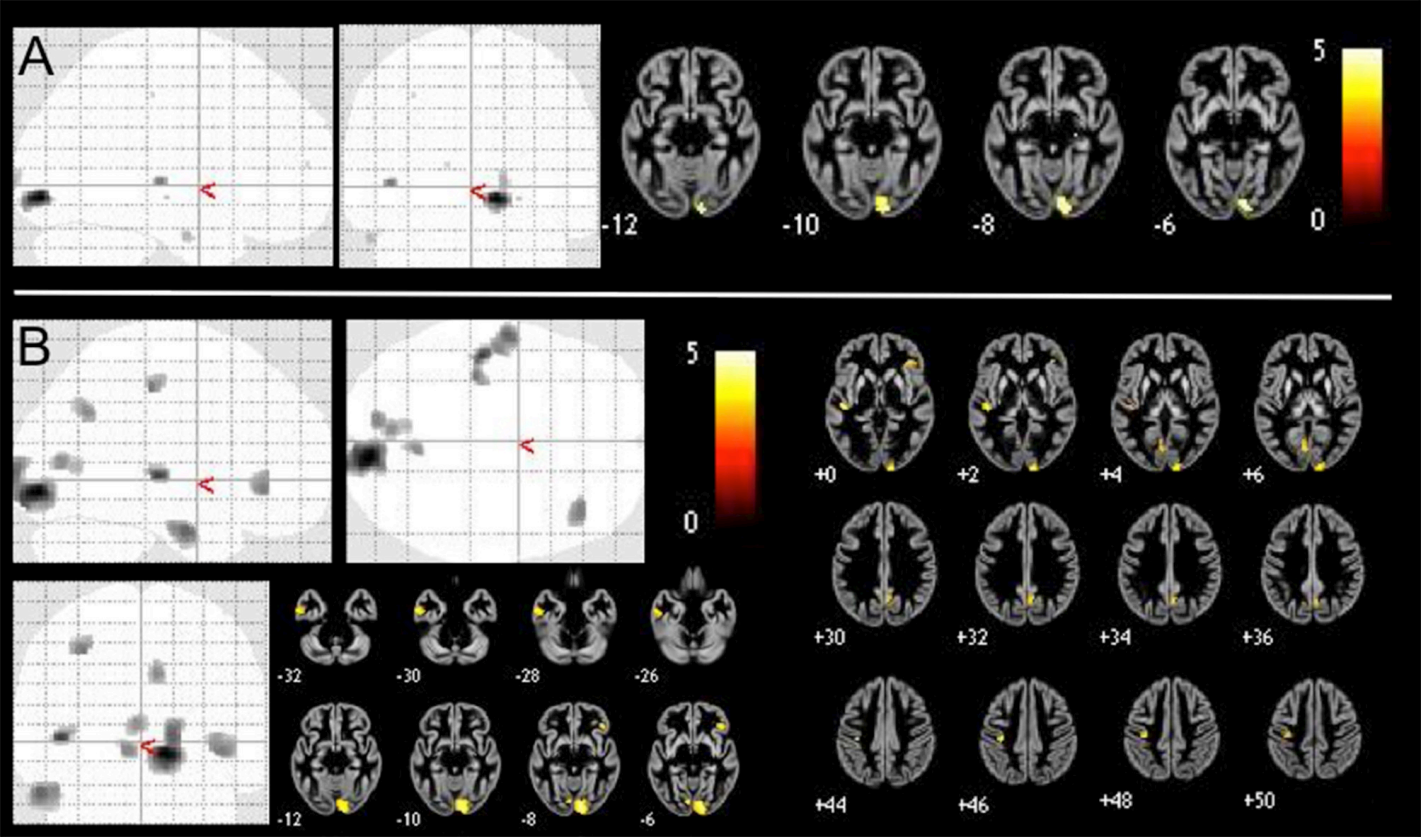
Positive correlations of GMV with SCL-90-R phobia subscale value in 409 healthy controls VBM-results are presented as maximum intensity projections (gray color indicates areas of significant positive correlations) and slice overlays on a gray matter average image of the whole sample yellow color indicates areas of significant positive correlation. We performed **(A)** brain-wide analysis with *p* < 0.05, FDR-corrected and **(B)** an exploratory brain-wide analysis with *p* < 0.001, uncorrected.

Our exploratory analysis at *p* < 0.001 (uncorrected) revealed positive correlations of GMV in addition to the above clusters in left precentral and post-central gyri and precuneus as well as right orbitofrontal cortex (OFC), insula, precuneus, and posterior cingulate gyrus. An overview of both results is given in Table 2 and presented in Figure 1.

**Table 2:** Overview of significant clusters of positive correlations of GMV with SCL-90-R phobia subscale values.

**Anatomical region**	**Co-ordinates of peak voxel**	***k***	***p (peak-level)***	***T***
**Main, FDR-corrected analysis**
Right lingual gyrus and bilateral calcarine sulcus	10; −87; −8	308	0.017	5.12
Left superior and middle temporal gyrus	−45; −22; 0	14	0.024	4.45
Right calcarine sulcus	15; −102; 0	30	0.036	4.19
Inferior and middle temporal gyrus	−56; −8; −28	22	0.037	4.16
**Exploratory, uncorrected analysis (exp.voxels/cluster**, ***k*** = **101.83)**
Right lingual gyrus, bilateral calcarine	10; −87; −8	1,190	0.0001	5.12
sulcus, right cuneus, and cerebellum (BA6)	15; −102; 0			4.19
Left superior and middle temporal gyri	−45; −22; 0	119	0.0001	4.45
Left middle and inferior temporal gyri	−56; −8; −28	434	0.0001	4.16
	−48; −14; −27			3.87
Left precentral and post-central gyri	−33; −27; 45	101	0.0001	4.01
Right OFC and insula	42; 33; −6	258	0.0001	3.92
Left lingual gyrus and calcarine sulcus	−9; −82; −6	124	0.0001	3.87
Bilateral precuneus	6; −62; 33	134	0.0001	3.82
Left lingual gyrus and calcarine sulcus	−4; −75; 8	203	0.0001	3.8
	−6; −68;4			3.45

*K, cluster size (voxel)*.

There were no negative correlations of gray matter with the SCL90R phobia subscale (neither at *p* < 0.05 FDR-corrected, nor *p* < 0.001 uncorr. levels).

While in our sample SCL-90-R phobic anxiety subscale was not significantly correlated with either age (*p* = 0.44, *r* = −0.038, two-tailed Pearson correlation) or gender (*p* = 0.638, *r* = −0.023, two-tailed Pearson correlation), we additionally reanalysed the data with those variables as nuisance variables. Overall, this confirmed our previous analysis with significant (*p* < 0.05, FDR-corrected) findings, in particular right lingual gyrus and calcarine sulcus, as well as left calcarine sulcus, superior, middle, and inferior temporal gyri.

## Discussion

In this study, we demonstrate a brain-wide pattern of GMV associations with minor/subclinical agoraphobic symptoms in non-clinical, psychiatrically healthy subjects. Overall, our findings are consistent with a putative neurobiological spectrum of anxiety, which manifests as an incremental effect of an anxiety dimension on brain structure. However, our study also shows some positive correlations with symptoms, thus suggesting that an overly simplified model of linear effects between phenotypes and their neural correlates is not likely to explain the complex relationship between phenotypes and brain structural variation. While there are only very few studies relating anxiety symptoms to brain morphology, we shall discuss our findings in comparison to case-control studies of agoraphobia and panic disorder.

A study focussing on trait anxiety (as determined by the T-AI measuring anxiety related trait personality) in 382 university students found a negative correlation with GMV in the right middle occipital gyrus (36). The authors argued that conscious-level visual processing might be impaired in persons with higher trait anxiety, but the lack a functional anatomical model as well as effects in the functional MRI part of the same study in this area limit further interpretation of those findings. In a study on social anxiety in 130 healthy adults social distress was correlated with the cortical volume of the right OFC and left amygdala, while social avoidance was correlated with the cortical volume of the right dorsolateral prefrontal cortex (DLPFC) (37). Also, a study in *n* = 320 adults showed positive associations of separation anxiety scores with increased amygdala GMV (38). In our own previous work, a VBM study of the Jena-1 sample (177 healthy subjects) using the general anxiety scale of the SCL-90-R, which reflects more general symptoms rather than those reflected in the phobia scale, we previously showed positive GMV correlations with subclinical symptoms of general anxiety in middle temporal gyrus, Rolandic operculum, middle cingulate gyrus, and precuneus bilaterally (26). Hence, the findings diverge in particular for the occipital cortical findings, which were only present in the current multi-center study of the agoraphobic anxiety scale.

Although there seems to be some overlap of findings in these studies of healthy subjects, the comparison suffers from the considerable heterogeneity of tools used to measure duration and quality of anxiety symptoms. To our knowledge, there is currently no other study on brain structural correlates of mild agoraphobic symptoms in healthy participants.

Nonetheless, alterations in insula, cingulate, and temporal cortices and OFC have robustly been shown in previous case-control studies of generalized anxiety disorder, specific phobia, social anxiety disorder, panic disorders, and agoraphobia (18, 20, 23). In the panic disorder/agoraphobia spectrum, several studies appear to converge on effects in the amygdala, ACC, insula, and lateral prefrontal cortex, but also for occipital brain areas. This indicates a partial overlap with our findings (although not for key regions like the ACC), and thus supports the notion of a biological continuum at least in some brain areas.

GMV reduction in the amygdala of patients with panic disorder was found in early studies (39–41), although not all studies (42), but it is unclear whether this is also present in agoraphobia (18). GMV alterations in insula and ACC were repeatedly replicated in patients with panic disorders, hinting at participation of these areas in the evaluation of (negative) emotional meaning of potentially distressing cognitive and interoceptive sensory information. It was also hypothesized that altered brain stem structures could mediate panic attacks (40). Temporal volume reductions in patients with panic disorder occurred mainly in right inferior and left superior temporal gyri. The same study also reported GMV reductions in right inferior and left superior frontal gyri, right precuneus and bilateral putamen (43). Also, decreased GMV was found in left medial OFC in patients with panic disorder and agoraphobia, but not without agoraphobia, suggesting the area especially involved in phobic avoidance of certain situations (44). Another study implicates a neurodevelopmental background for volume reduction of the posterior region of the OFC in patients with panic disorder, since the area receives multisensory input (45, 46).

A particularly noteworthy result of our study is the effect of phobic anxiety in posterior, mostly occipital, cortices, which in fact survived correction for multiple comparisons. Lingual cortex changes (reduction in patients compared to controls) was found in one previous study, which included panic disorder patients with co-morbid depression, so specificity of findings remains unclear (47). Functional MRI reports detected lowered activation in fusiform and inferior occipital gyri, the calcarine sulcus, cerebellum, and cuneus, as well as higher activity in precuneus in patients with panic disorder and agoraphobia compared with healthy controls, when confronted with emotionally neutral pictures of faces and places. The authors suggest higher anxiety-related arousal in those patients (22). A recent functional MRI study has, however, also suggested that some of these effects are seen across multiple anxiety disorders, hence again questioning specificity (21). Other functional changes of in panic disorder include metabolic changes studied with MR spectroscopy. Patients with panic disorder exhibit a 22% reduction in total occipital cortex GABA concentration (GABA plus homocarnosine) (48). Interestingly, occipital regions of visual processing appeared to be enlarged in patients with social anxiety disorder, and have been linked to abnormal emotional information processing (49), although this finding has not been replicated in a more recent study (50). While a recent meta-analysis of anxiety and mood disorders has associated aberrant activations in occipital cortices with emotional experiences (51), it appears that our understanding of these changes is still very incomplete and necessitates further study and model development.

Heterogeneity in our samples might be related to liability for agoraphobia as well as resilience toward the full expression of a clinical phenotype. Subjects with particular brain structural features (e.g., in the insula and other regions) might be particularly prone not only to subthreshold symptoms, but might have a higher incidence of clinical anxiety disorders. However, it remains unclear how this might interact with protective factors, which themselves might be associated with regional gray matter enlargement and thus prevent the gray matter reduction seen in clinically manifest disorders. Since we did not have follow-up data to study subjects who later might have developed agoraphobia or another anxiety disorder, our analysis remains cross-sectional.

We conclude that even temporary agoraphobic symptoms, which might be related to stressful life episodes, have an impact on cortical volume of healthy people in part similar to patients. Additional areas like primary visual cortex also correspond with functional and metabolic findings in patients in some anxiety disorders, suggesting these areas importance for early pathogenesis. Investigating a homogeneous sample of healthy young adults possibly enables detection of areas otherwise statistically not significant when comparing patients to controls without diagnosis but an uncharted amount of mild agoraphobic symptoms. Interestingly, many correlations in our study were positive, echoing recent findings in subclinical symptoms, while in clinically manifest disorders, categorical effects mostly show volume reductions in patients. This might be explained by different factors. We propose that the association between symptoms and regional gray matter, when studied across the full spectrum (from minor sub-clinical to increased sub-clinical, to sub-threshold clinical, and finally manifest clinical expressions of the phenotype) might be non-linear. For example, if the association was best represented by an “inverted U shape” curve, one would reasonably expect positive (linear) correlation when restricting the analysis to the lower end (non-clinical) spectrum, while correlations in the higher end of the spectrum (i.e., clinically manifest disorder) would show negative correlations, i.e., gray matter loss with increasing severity of symptoms. However, our results might also be related to uncontrolled confounders sharing variance with the SCL90R score.

The brain structural variations identified here might not only relate to liability for agoraphobia; indeed, they might also relate to compensatory changes on a structural level, which ultimately result in a non-clinical phenotype. Studies of subclinical symptoms are, however, still scarce and often limited to small samples. Yet, they hold a potential to better understand the interactions of liability or risk vs. resilience, and the neural sequelae of this dynamic process, which might result in the expression of a disease phenotype vs. temporal expression of subclinical symptoms. Given the recent importance placed in identification of biotypes of anxiety disorders (52), our findings argue for also considering the full spectrum of psychopathology and a spectrum approach, which takes into account both categorical as well as dimensional expressions of symptoms in patients as well as the general population.

## Author contributions

BB, IN, and PB planned the study. BB and IN wrote the first draft of the manuscript. BB, LS, RS, and MB collected the data. BB and CG conducted the VBM analysis. All authors contributed their ideas to the final version of the manuscript.

### Conflict of interest statement

The authors declare that the research was conducted in the absence of any commercial or financial relationships that could be construed as a potential conflict of interest.
